# Extracellular Vesicles from Adipose-Derived Mesenchymal Stem Cells Downregulate Senescence Features in Osteoarthritic Osteoblasts

**DOI:** 10.1155/2017/7197598

**Published:** 2017-11-05

**Authors:** Miguel Tofiño-Vian, Maria Isabel Guillén, María Dolores Pérez del Caz, Miguel Angel Castejón, Maria José Alcaraz

**Affiliations:** ^1^Instituto Interuniversitario de Investigación de Reconocimiento Molecular y Desarrollo Tecnológico (IDM), Universitat Politècnica de València, Universitat de València, Av. Vicent A. Estellés s/n, Burjasot, 46100 Valencia, Spain; ^2^Department of Pharmacy, Cardenal Herrera-CEU University, Ed. Ciencias de la Salud, Alfara, 46115 Valencia, Spain; ^3^Department of Burn and Plastic Surgery, La Fe Polytechnic University Hospital, 46026 Valencia, Spain; ^4^Department of Orthopaedic Surgery and Traumatology, De la Ribera University Hospital, Alzira, 46600 Valencia, Spain

## Abstract

Osteoarthritis (OA) affects all articular tissues leading to pain and disability. The dysregulation of bone metabolism may contribute to the progression of this condition. Adipose-derived mesenchymal stem cells (ASC) are attractive candidates in the search of novel strategies for OA treatment and exert anti-inflammatory and cytoprotective effects on cartilage. Chronic inflammation in OA is a relevant factor in the development of cellular senescence and joint degradation. In this study, we extend our previous observations of ASC paracrine effects to study the influence of conditioned medium and extracellular vesicles from ASC on senescence induced by inflammatory stress in OA osteoblasts. Our results in cells stimulated with interleukin- (IL-) 1*β* indicate that conditioned medium, microvesicles, and exosomes from ASC downregulate senescence-associated *β*-galactosidase activity and the accumulation of *γ*H2AX foci. In addition, they reduced the production of inflammatory mediators, with the highest effect on IL-6 and prostaglandin E_2_. The control of mitochondrial membrane alterations and oxidative stress may provide a mechanism for the protective effects of ASC in OA osteoblasts. We have also shown that microvesicles and exosomes mediate the paracrine effects of ASC. Our study suggests that correction of abnormal osteoblast metabolism by ASC products may contribute to their protective effects.

## 1. Introduction

Osteoarthritis (OA) is the most prevalent joint disease and a leading cause of pain and disability in the aging population. OA affects the whole joint leading to cartilage degradation, synovitis, formation of osteophytes, and bone sclerosis. Several studies have demonstrated that bone metabolism is dysregulated in OA and may contribute to the onset and/or progression of this condition [1, 2]. Therefore, the modification of the abnormal metabolism of bone cells may lead to novel approaches for OA treatment [3].

It is known that osteoblasts participate in the regulation of cartilage metabolism and bone remodeling in OA [4]. In particular, subchondral osteoblasts from OA patients show altered phenotypic characteristics [5, 6]. These cells are able to induce a phenotypic shift in OA chondrocytes towards the hypertrophic state [7] as well as the production of matrix metalloproteinases and the inhibition of aggrecan synthesis [8] which play an important role in cartilage degradation [9]. In addition, sites more distal to the joint articular surface show more rigid trabecular bone structure and lower mineralization related to an altered state of trabecular bone remodeling [10].

Proinflammatory cytokines are elevated in synovial fluid, synovial membrane, cartilage, and subchondral bone and have synergistic effects on inflammation, cartilage degradation, and bone remodeling in OA and diseases characterized by bone loss [11–13]. Interleukin- (IL-) 1*β* and tumor necrosis factor-*α* (TNF*α*) are thought to enhance osteoclastogenesis and bone resorption but they inhibit osteoblast differentiation and bone formation [13, 14]. Additionally, chronic inflammation can lead to cellular senescence in OA [15]. As a model of inflammatory stress, IL-1*β* stimulation of OA osteoblasts results in metabolic changes and the production of inflammatory and catabolic mediators as well as senescence features [16].

Novel therapeutic approaches for OA are being investigated as there is no pharmacological treatment able to modify the joint structural alterations. Some examples can be the injection of autologous and allogeneic mesenchymal stem cells or the differentiation into cartilage using scaffolds (reviewed in [17]). A wide range of evidence has shown the interest of adipose-derived mesenchymal stem cells (ASC) in tissue regeneration and cytoprotection. For instance, the administration of ASC into the knee joint inhibited synovial activation and prevented cartilage damage in experimental OA [18, 19]. The cytoprotective and anti-inflammatory properties of ASC in human chondrocytes and experimental OA may be mediated by paracrine effects [20–22] which are also responsible for the inhibition of senescence in OA chondrocytes [23].

There is an increasing interest to know the properties of extracellular vesicles as novel ways of cellular communication [24]. The conditioned medium (CM) of ASC contains extracellular vesicles, mainly microvesicles (MV), and exosomes (EX), which may contribute to the paracrine effects of ASC. In this study, we have extended our previous observations in OA chondrocyte senescence [23] to investigate the contribution of extracellular vesicles to the paracrine effects of ASC on the cellular stress leading to senescence in OA osteoblasts.

## 2. Materials and Methods

### 2.1. Adipose-Derived Mesenchymal Stem Cells

ASC were isolated from the adipose tissue of 8 abdominoplasty-undergone healthy donors (2 men and 6 women, aged 54.4 ± 14.1 years, mean ± SEM). The experimental design was approved by the Institutional Ethical Committees (University of Valencia and La Fe Polytechnic University Hospital, Valencia, Spain). Samples were obtained from donors after they provided informed consent according to the Helsinki Declaration of 1975, as revised in 2013.

Samples were washed with phosphate-buffered saline (PBS) and minced and digested at 37°C for 1 h with 2% of type I collagenase (Gibco, Life Technologies, Madrid, Spain). Tissue remains were filtered through a 100 μm cell strainer (BD Biosciences Durham, NC, USA). Cells were then washed with DMEM/HAM F12 (Sigma-Aldrich, St. Louis, MO, USA) containing penicillin and streptomycin (1%), seeded onto tissue culture flasks (1-2 × 10^6^ cells/mL) in DMEM/HAM F12 medium with penicillin and streptomycin (1%), supplemented with 15% extracellular vesicle-free human serum, and incubated at 37°C in a 5% CO_2_ atmosphere. Human serum was obtained from whole-blood donations of AB-blood-group-typed donors according to the criteria of Valencia Transfusion Centre. To eliminate the extracellular vesicle fraction, serum was centrifuged during 18 h at 120,000 ×g and 4°C using a SW-28 swinging-bucket rotor (Beckman Coulter, CA, USA). When cells reached semiconfluence, culture plates were washed and the ASC phenotype confirmed by flow cytometry (Flow Cytometer II, BD Biosciences, San Jose, CA, USA) using specific antibodies, anti-CD105-PE, antiCD90PerCP-eFluo 710, anti-CD34APC (eBioscience Inc., San Diego, CA, USA), and anti-CD45-PE (BD Pharmingen), and measuring cellular viability with propidium iodide. Finally, conditioned medium (CM) was collected from ASC culture cells at passage 0 every 48 h of culture. It was pooled, centrifuged, and stored in sterile conditions at −80°C prior to further use.

### 2.2. Isolation of Extracellular Vesicles

Vesicles were obtained from the CM of ASC using a filtration/centrifugation-based protocol. Cellular debris was eliminated by centrifugation at 300 ×g for 10 min. Vesicles were then collected from the supernatant through differential centrifugation steps. CM was filtered through 800 nm filter (Merck, Darmstadt, Germany) and centrifuged at 12,200 ×g for 20 min at 4°C to pellet microvesicles. Then, supernatants were filtered through a 200 nm filter (Merck, Darmstadt, Germany) and centrifuged at 100,000 ×g for 90 min at 4°C. Pellets were washed once with sterile PBS, resuspended in 15 *μ*L of PBS, and stored at −80°C until further use.

### 2.3. Tunable Resistive Pulse Sensing

Extracellular vesicle preparations were analyzed by Tunable Resistive Pulse Sensing (TRPS) using a qNano instrument (IZON Science Ltd., Oxford, UK) as previously described [25]. Briefly, NP100 and NP300 nanopore membranes were used to measure the samples of EX and MV, respectively. At least 500 events/sample were counted. Calibration was performed using calibration beads SKP200 and SKP400, provided by the manufacturer (IZON Science Ltd.).

### 2.4. Transmission Electron Microscopy

Preparation of samples for transmission electron microscopy (TEM) was performed by the Microscopy Service (SCSIE, University of Valencia). LR-white resin inclusion was performed. Samples were filtered in resin and polymerized at 60°C for 48 h. Ultrathin slices (60 nm) were made with a diamond blade (DIATOME, Hartfield, USA) in eyelet grilles in a UC6 Ultracut (Leica, Wetzlar, Germany) and stained with uranyl acetate 2% for 25 min and lead citrate 3% for another 12 min prior to visualization in Jeol-1010 (JEOL Ltd. Tokyo, Japan) at 60 kV. Images were acquired with a digital camera MegaView III with Olympus Image Analysis Software (Olympus, Tokyo, Japan).

### 2.5. OA Osteoblasts

Knee specimens were obtained from patients with advanced OA diagnosed (21 women and 9 men, aged 68.4 ± 9.6 years, mean ± SEM) undergoing total knee joint replacement. Diagnosis was based on clinical and radiological evaluation. The experimental design was approved by the institutional ethical committees (University of Valencia and La Fe Polytechnic University Hospital, Valencia, Spain). Samples were obtained from donors after they provided informed consent according to the Helsinki Declaration of 1975, as revised in 2013.

Trabecular bone samples were obtained from the femoral condyles and tibial plateaus, cut into small pieces, and subjected to enzymatic digestion with 1 mg/mL of collagenase type IA (Sigma-Aldrich) at 37°C in DMEM/HAM F-12 (Sigma-Aldrich), containing penicillin and streptomycin (1%) for 2 h. The digested tissue was cultured in osteoblast medium (Promocell, Labclinics S.A., Barcelona, Spain) in a humidified incubator with 5% CO_2_ at 37°C. This medium was replaced twice a week. When cells were at 70% of confluence, bone fragments were removed and cells were allowed to grow until confluent. Cell phenotype was characterized by flow cytometry analysis using a Becton Dickinson FACSCanto II cytometer (BD, Franklin Lakes, NJ) and specific antibodies as previously reported [26]. For cell stimulation and treatment, subconfluent osteoblasts were incubated for 24 h in DMEM/HAM F12 (Sigma-Aldrich) containing penicillin and streptomycin (1%), supplemented with 15% extracellular vesicle-free human serum, and stimulated with IL-1*β* (10 ng/mL) in the presence or absence of MV (3.6 × 10^7^ particles/mL), EX (7.2 × 10^7^ particles/mL), or CM for 24 h (or 7 days for senescence-associated *β*-galactosidase activity (SA-*β*-Gal) experiments). These concentrations are in the range of those present in CM used in the same experiments.

### 2.6. Senescence-Associated *β*-Galactosidase Activity

Osteoblasts were seeded at 20 × 10^3^ cells/well in Lab-Tek chambers (Thermo Scientific, Rochester, NY, USA), then stimulated with IL-1*β* (10 ng/mL), and treated with MV (3.6 × 10^7^ particles/mL) or EX (7.2 × 10^7^ particles/mL) or CM (0.2 mL) for 7 days. SA-*β*-Gal activity was measured using the cellular senescence assay kit from Cell Biolabs (San Diego, CA) in its fluorometric format. Briefly, cells were washed with cold PBS and lysed during 5 minutes at 4°C. Lysates were centrifuged and supernatant was collected as cell lysate. After transfer to fluorescence 96-well plates, lysates were incubated in the presence of assay buffer during 1 h at 37°C. Reaction was stopped and fluorescence was measured at 360 nm (excitation)/465 nm (emission) in a Victor3 microplate reader (PerkinElmer España, Madrid, Spain).

### 2.7. Immunofluorescence Assay for *γ*H2AX Foci

Osteoblasts were seeded at 20 × 10^3^ cells/well in Lab-Tek chambers (Thermo Scientific, Rochester, NY, USA), then stimulated with IL-1*β* (10 ng/mL), and treated with MV (3.6 × 10^7^ particles/mL) or EX (7.2 × 10^7^ particles/mL) or CM (0.2 mL) for 24 h. All cells were fixed with 4% formaldehyde in PBS for 30 min at 4°C and blocked with 5% normal goat serum and 0.3% Triton X-100 in PBS for 60 min at room temperature. Osteoblasts were further incubated with phospho-histone H2AX (Ser139) antibody (Cell Signaling Technology, Beverly, MA, USA) overnight at 4°C. Finally, cells were incubated with FITC-conjugated goat antirabbit IgG (R&D Biosystems, Abingdon, UK), mounted in Prolong Gold antifade reagent with DAPI, and examined under a confocal microscope (Olympus FV1000, Tokyo, Japan).

### 2.8. Enzyme-Linked Immunosorbent Assay

Osteoblasts were stimulated with IL-1*β* (10 ng/mL) in the presence or absence of MV (3.6 × 10^7^ particles/mL), EX (7.2 × 10^7^ particles/mL), or CM (1 mL) for 24 h. Supernatants were harvested, centrifuged, and frozen at −80°C until analysis. In order to measure the levels of 4-hydroxy-nonenal (HNE) proteins, cells were lysed with 1% Triton X-100, 1% deoxycholic acid, 20 mM NaCl, and 25 mM Tris, and pH 7.4 buffer. Lysates were centrifuged at 4°C for 10 min at 10,000 ×g. Then, 4-HNE-modified proteins were measured with the Cell Biolabs ELISA kit (San Diego, CA, USA) with sensitivity of 1.56 *μ*g/mL. TNFα, IL-6, and IL-10 were measured in supernatants with ELISA kits from eBioscience (San Diego, CA, USA) with a sensitivity of 4.0 pg/mL for TNF*α* and IL-6 and 2.0 pg/mL for IL-10.

### 2.9. Determination of Prostaglandin E_2_

Osteoblasts were stimulated with IL-1*β* (10 ng/mL) in the presence or absence of MV (3.6 × 10^7^ particles/mL), EX (7.2 × 10^7^ particles/mL), or CM (1 mL) for 24 h. Supernatants were used to measure prostaglandin E_2_ (PGE_2_) by radioimmunoassay as previously described [27] using a Victor3 microplate reader (PerkinElmer España, Madrid, Spain).

### 2.10. Mitochondrial Membrane Potential

Osteoblasts were stimulated with IL-1*β* (10 ng/mL) in the presence or absence of MV (3.6 × 10^7^ particles/mL), EX (7.2 × 10^7^ particles/mL), or CM (1 mL) for 24 h. Then, mitochondrial transmembrane potential (Δ*ψ*m) was assessed with the JC-1 probe (5,5′,6,6′-tetrachloro-1,1′,3,3′-tetraethyl-benzamidazolylcarbocyanine iodide, Thermo Scientific, Rochester, NY, USA). This lipophilic membrane-permeant cation exhibits potential-dependent accumulation in mitochondria, indicated by a fluorescence emission shift from ~525 nm (monomeric form) to ~590 nm (aggregated form). Cell cultures were trypsinized, resuspended in 1 mL of PBS, and incubated with 10 *μ*g/mL of JC-1 dye for 15 min at 37°C and 5% CO_2_. Both red and green fluorescence emissions were analyzed by flow cytometry using an excitation wavelength of 488 nm and observation wavelengths of 530 nm for green fluorescence and 585 nm for red fluorescence and a Becton Dickinson FACSCanto II cytometer (BD, Franklin Lakes, NJ, USA).

### 2.11. Statistical Analysis

The data were analyzed by one-way analysis of variance (ANOVA) followed by Sidak's posttest using the GraphPad Prism 7.0 software (GraphPad Software, La Jolla, CA, USA). A *P* value of less than 0.05 was considered to be significant.

## 3. Results

### 3.1. Characterization of MV and EX from ASC

**Figure 1:**
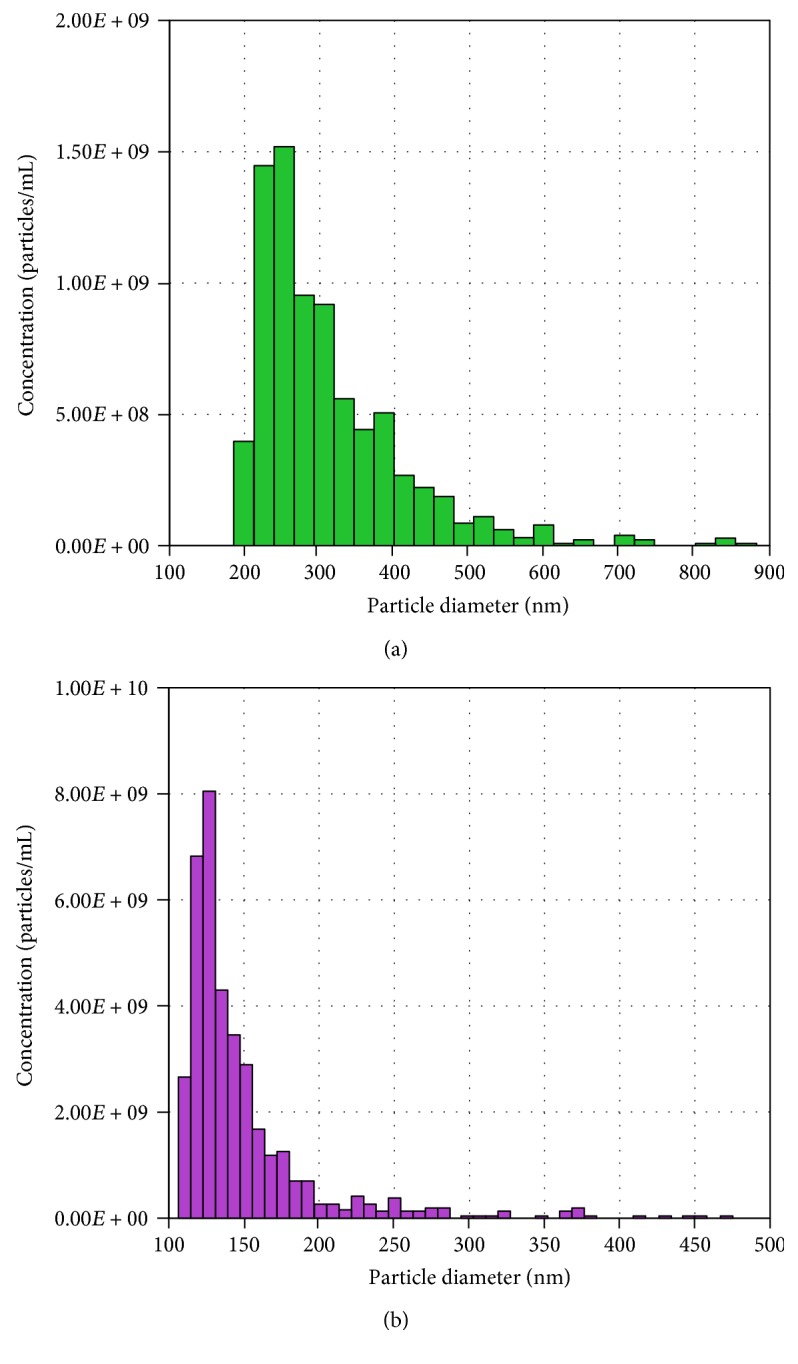
Characterization of MV and EX isolated from CM. Representative TRPS analysis of MV (a) and EX (b).

MV and EX fractions were isolated as indicated in Materials and Methods. TRPS analysis indicated a mean diameter of 316 nm and 115 nm and a concentration of 8 × 10^9^ and 3.8 × 10^10^ particles/mL for MV and EX, respectively.  Figure 1 shows a representative TRPS analysis of MV (a) and EX (b) fractions. The morphology of MV and EX was studied by TEM (data not shown).

### 3.2. SA-*β*-Gal Activity Induced by IL-1*β* in Human OA Osteoblasts

**Figure 2:**
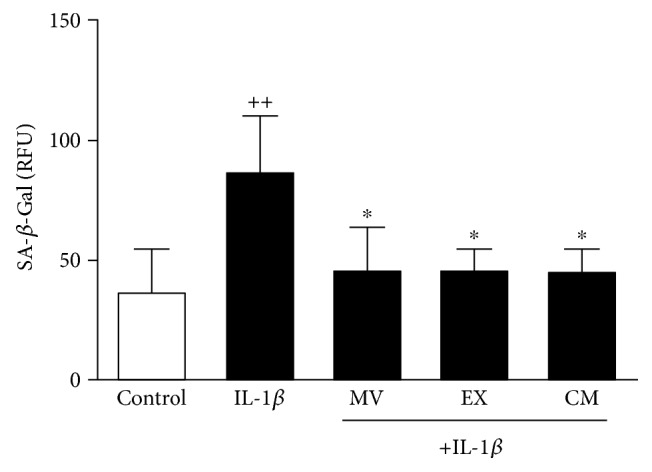
SA-*β*-Gal activity in OA osteoblasts. Cultures were treated with IL-1*β* alone or in combination with MV, EX, or CM for 7 days. SA-*β*-Gal activity was measured by using the cellular senescence assay kit (Cell Biolabs) and expressed as relative fluorescence units (RFU). Results show mean ± SD from 4 separate experiments with cells from separate donors. ^++^*P* < 0.01 compared to control (nonstimulated cells); ^∗^*P* < 0.05 compared to IL-1*β*.

We examined SA-*β*-Gal activity in OA osteoblasts for the effects of CM and extracellular vesicles on this marker of cellular senescence.  Figure 2 shows that IL-1*β* stimulation for 7 days enhanced SA-*β*-Gal activity by 57% with respect to control (nonstimulated cells). We found that treatment with MV, EX, or CM resulted in similar effects with a significant reduction of this activity by 48% with respect to IL-1*β*.

### 3.3. *γ*H2AX Foci Accumulation

**Figure 3:**
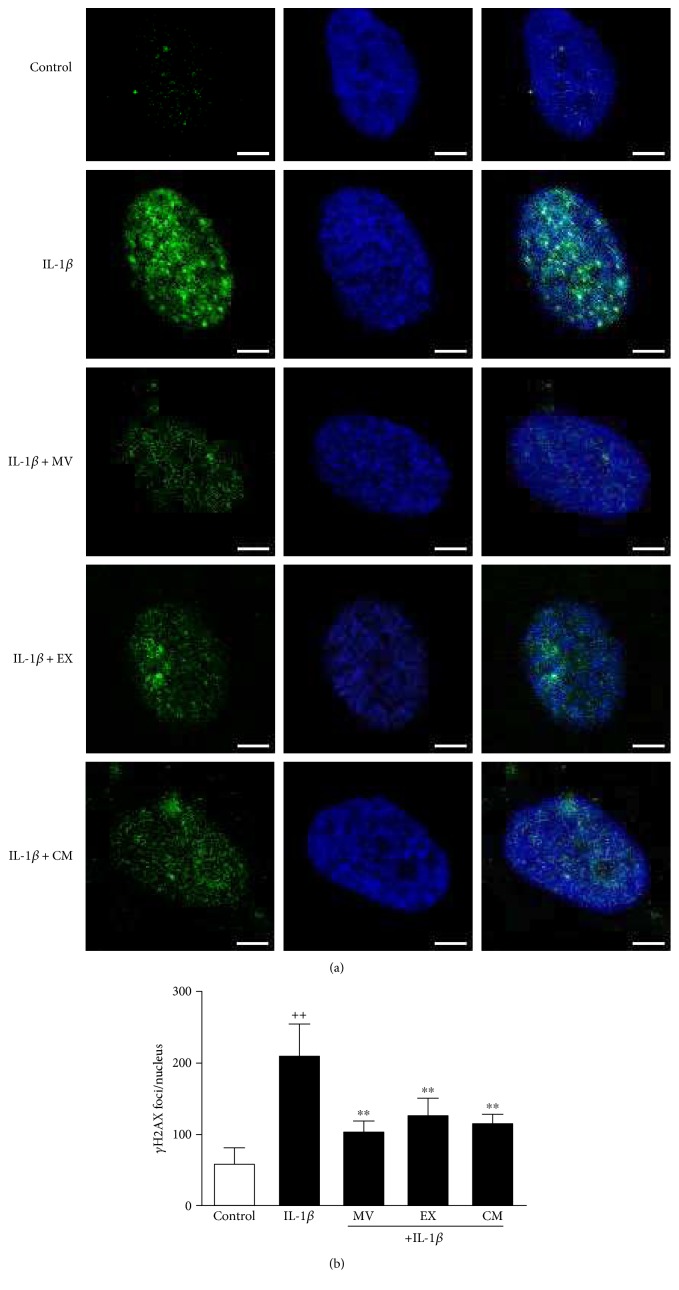
Immunofluorescence analysis of *γ*H2AX foci accumulation. (a) Representative images. *γ*H2AX foci (green, FITC fluorescence) and nuclei were stained with DAPI (blue). (b) Number of *γ*H2AX foci per nucleus. Cultures were treated with IL-1*β* alone or in combination with MV, EX, or CM for 24 h. Bar = 5 *μ*m. Results are expressed as mean ± SD from 3 separate experiments with cells from separate donors. ^++^*P* < 0.01 compared to control (nonstimulated cells); ^∗∗^*P* < 0.01 compared to IL-1*β*.

The presence of phosphorylated histone H2AX indicates DNA damage and correlates with age [28]. To assess the effect of CM and extracellular vesicles, *γ*H2AX foci were quantified in nuclei. The immunofluorescence analysis showed that *γ*H2AX foci were increased in the presence of IL-1*β* for 24 h by 70% compared with control (nonstimulated cells) (Figures 3(a) and 3(b)). The amount of *γ*H2AX foci per nucleus was significantly reduced by treatment with MV (46%), EX (44%), or CM (30%).

### 3.4. Production of Proinflammatory and Anti-Inflammatory Mediators

**Figure 4:**
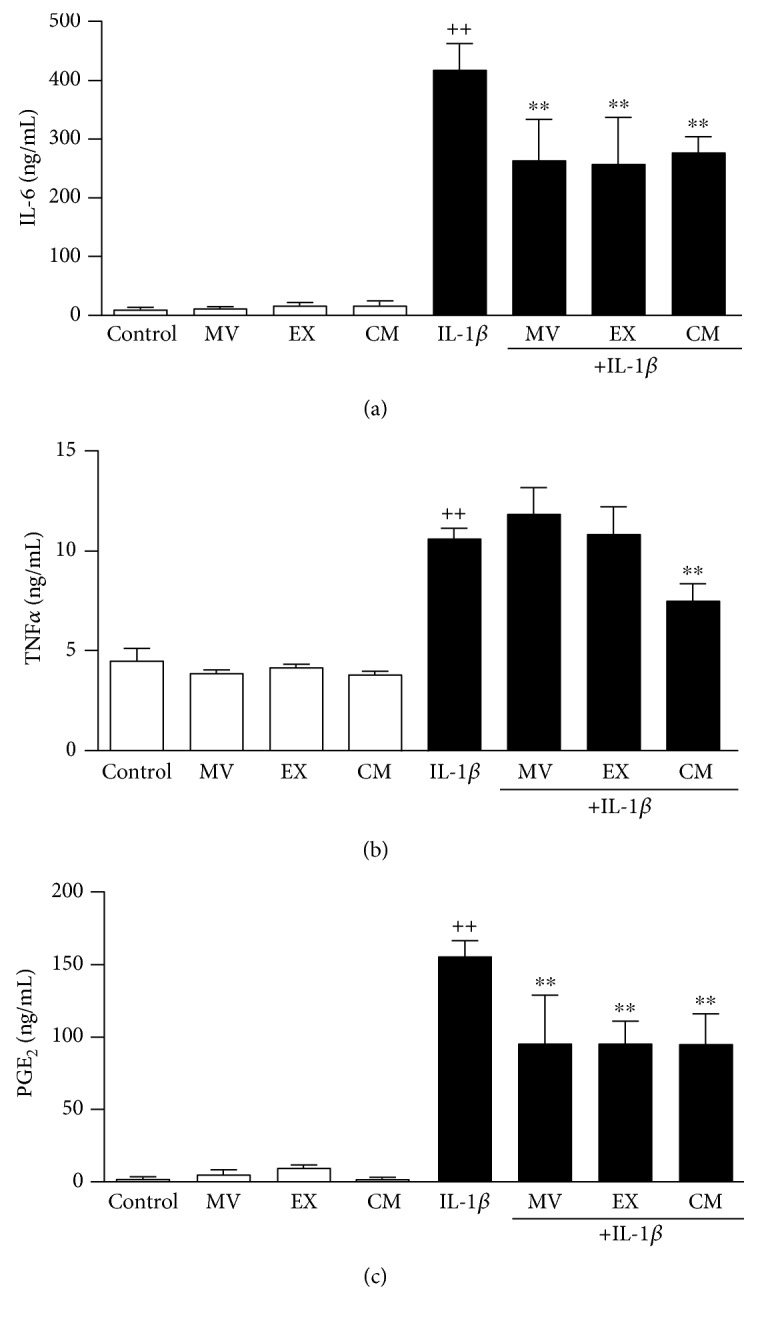
Release of inflammatory mediators. IL-6 (a) and TNF*α* (b) were measured by ELISA; PGE_2_ (c) was measured by radioimmunoassay in cell culture supernatants of OA osteoblasts. Cultures were treated with IL-1*β* and/or MV, EX, or CM for 24 h. Results are expressed as mean ± SD from 4 separate experiments with cells from separate donors. ^++^*P* < 0.01 compared to control (nonstimulated cells); ^∗∗^*P* < 0.01 compared to IL-1*β*.

**Figure 5:**
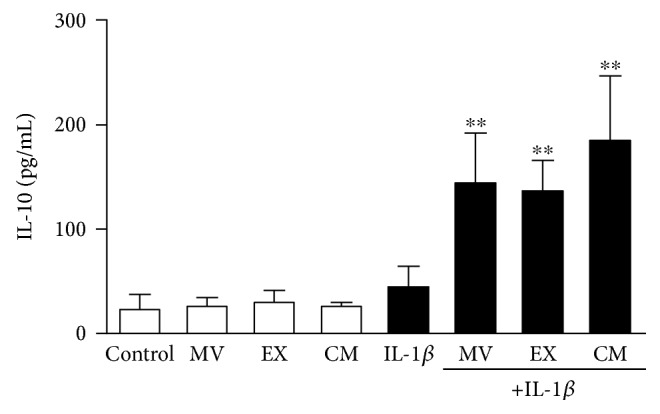
Release of IL-10 by OA osteoblasts. IL-10 was measured by ELISA in cell culture supernatants. Cultures were treated with IL-1*β* and/or MV, EX, or CM for 24 h. Results are expressed as mean ± SD from 5 separate experiments with cells from separate donors. ^∗∗^*P* < 0.01 compared to IL-1*β*.

Inflammation is involved in cellular senescence and OA. We have determined the production of key proinflammatory mediators in OA osteoblasts. After 24 h of incubation, IL-1*β* strongly induced the production of proinflammatory cytokine IL-6 and the eicosanoid PGE_2_ while TNF*α* levels were enhanced to a lower extent (Figure 4). Treatment with MV, EX, or CM did not affect the basal release of these mediators. Nevertheless, MV, EX, and CM significantly reduced IL-6 and PGE_2_, and CM also decreased TNF*α* levels in cells stimulated with IL-1*β*. In addition, the anti-inflammatory cytokine IL-10 was measured in this system. As shown in Figure 5, after MV, EX, or CM treatment, the levels of IL-10 significantly increased by more than threefold after 24 h of incubation in the presence of IL-1*β*.

### 3.5. Oxidative Stress

**Figure 6:**
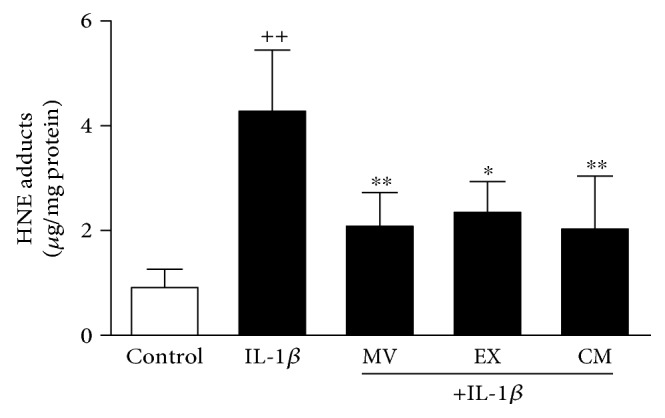
Quantification of HNE-protein adducts in OA osteoblasts. HNE-protein adducts were measured by ELISA in cellular extracts. Cultures were treated with IL-1*β* alone or in combination with MV, EX, or CM for 24 h. Results are expressed as mean ± SD from 4 separate experiments with cells from separate donors. ^++^*P* < 0.01 compared to control (nonstimulated cells); ^∗^*P* < 0.05 and ^∗∗^*P* < 0.01 compared to IL-1*β*.

As oxidative stress is a key process in the induction of cellular senescence [29], we next investigated the effects of CM, MV, and EX on protein modification by oxidative stress. As shown in Figure 6, IL-1*β* induced the production of oxidative stress leading to the accumulation of HNE-modified proteins in OA osteoblasts. We observed a significant reduction (by 50%) in the amount of HNE-protein adducts measured in cells treated with CM, MV, or EX.

### 3.6. Mitochondrial Membrane Potential

**Figure 7:**
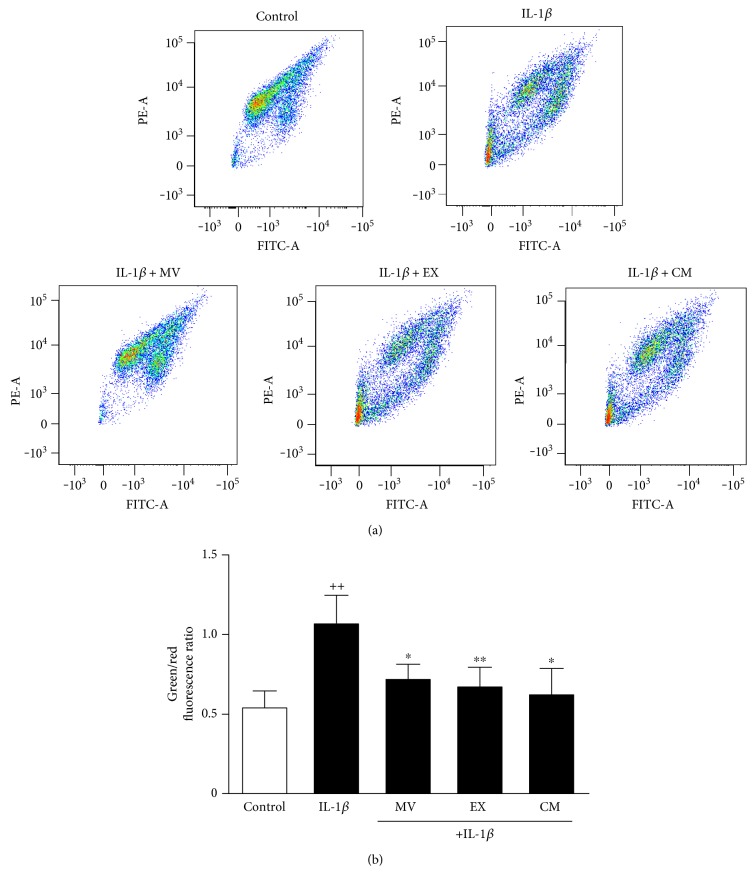
Analysis of mitochondrial membrane potential in OA osteoblasts. Analysis was performed by flow cytometry using the probe JC-1. Representative images (a); green/red fluorescence ratio (b). Cultures were treated with IL-1*β* alone or in combination with MV, EX, or CM for 24 h. Results are expressed as mean ± SD from 3 separate experiments with cells from separate donors. ^++^*P* < 0.01 compared to control (nonstimulated cells); ^∗^*P* < 0.05 and ^∗∗^*P* < 0.01 compared to IL-1*β*.

To measure changes in the mitochondrial membrane potential (∆*Ψ*), we have used the probe JC-1. Mitochondrial depolarization is indicated by a decrease in the red/green fluorescence intensity ratio. Incubation of OA osteoblasts with IL-1*β* increased the green/red ratio by twofold indicating a lowering of the mitochondrial membrane potential (Figure 7(a) and 7(b)). Treatment with CM, MV, or EX significantly restored the mitochondrial membrane potential.

## 4. Discussion

Multiple types of stress can lead to premature cellular senescence. It has been proposed that low-grade chronic inflammation during aging and associated pathologies can lead to oxidative stress and cell alteration-driving senescence. Therefore, oxidative stress induces telomere-independent senescence leading to cell dysfunction [30]. Senescent cells develop a senescence-associated secretory phenotype with production of cytokines such as IL-6, growth factors, or matrix metalloproteinases which are mediators of complex autocrine and paracrine effects leading to phenotypic changes in nearby cells and alterations of tissue microenvironment [31]. Therefore, the accumulation of senescent cells with aging results in tissue or organ dysfunction. In support of this theory, it has been demonstrated that elimination of senescent cells in mice delays age-related pathologies [32].

There is increasing evidence that chronic inflammation-related senescence and aging may contribute to the development of OA [33]. The majority of studies of cellular senescence in OA have focused on chondrocytes. Chondrocyte senescence has been detected in OA cartilage [34] where the accumulation of cells could contribute to tissue destruction [35, 36]. ASC may offer new therapeutic approaches to regulate premature senescence. Recently, we have reported that ASC and CM inhibit senescence in OA chondrocytes [23]. In the current work, we have demonstrated the paracrine effects of ASC to downregulate senescence features induced by inflammatory stress in OA osteoblasts as well as the relevant contribution of MV and EX.

Joint tissues release proinflammatory cytokines in response to a wide variety of agents leading to mitochondrial changes, increased synthesis of reactive oxygen species (ROS), and DNA alterations which can induce premature senescence. In osteoblasts, cellular senescence is an important mechanism of age-related dysfunction which causes bone loss [37]. Aging bone shows a reduced ability of response against mechanical stress linked to some characteristics such as intralacunar hypermineralization and lower osteocyte lacunar density [38] which are also present in OA [39]. Subchondral bone alterations and cartilage degeneration are important processes during OA progression [40]. Interestingly, transplantation of senescent fibroblasts into the knee joint region of mice induces an inflammatory response and alterations in cartilage and bone resembling OA [41].

Proinflammatory and catabolic mediators produced by subchondral bone may contribute to cartilage and bone changes. It is considered that osteoblast cytokines can transmit the subchondral bone plate and calcified cartilage and communicate with chondrocytes [42]. Therefore, osteoblasts produce IL-6 which regulates the balance of bone resorption and formation during bone remodeling and can promote matrix degradation directly in both bone and cartilage [43]. We have demonstrated the paracrine anti-inflammatory effects of ASC on OA osteoblasts, with downregulation of IL-6 and TNF*α*. In addition, our results indicate that MV and EX could be the mediators of ASC paracrine effects on IL-6 which is the inflammatory marker showing the strongest association with age-related disease and fragility [33]. In contrast, MV and EX did not significantly reduce the levels of TNF*α* suggesting that soluble mediators present in CM may be the factors responsible for the regulation of this cytokine. The high levels of PGE_2_ produced in our model of inflammatory stress were also reduced by CM and extracellular vesicles. The production of this eicosanoid is enhanced during cellular senescence in human fibroblasts [44]. Concerning bone metabolism, PGE_2_ stimulates bone formation at low concentrations but it may be inhibitory at high concentrations [45, 46] and this eicosanoid may be a mediator of osteoclastogenesis induced by IL-6 [47]. In addition, PGE_2_ may be an enhancing factor for IL-6 production in human osteoblasts [48]. Therefore, our results suggest that a decrease in PGE_2_ production contributes to the anti-inflammatory and antisenescence effects of ASC and it may help to counteract the consequences of chronic inflammation on bone metabolism. In addition, we have shown that CM, MV, and EX from ASC enhance the production of the anti-inflammatory cytokine IL-10 in the presence of IL-1*β* which may prolong the downregulation of the inflammatory response as this cytokine inhibits the production of ROS and proinflammatory cytokines by macrophages [49, 50] and PGE_2_ by OA synovial fibroblasts [51]. This effect of CM, MV, and EX on IL-10 is in line with that reported for CM in OA chondrocytes [52]. Of note, IL-10 has been proposed as a treatment option for inflammation-related bone loss [53].

Chronic oxidative stress related to aging or mechanical stress may lead to cellular senescence in joint tissues [54] and age-related alterations in osteoblast differentiation and function [37, 55]. The majority of ROS are produced by the mitochondria as a consequence of oxidative phosphorylation which generates a potential energy for protons (Δ*Ψ*) across the mitochondrial inner membrane. ROS generated within the mitochondria can damage mitochondrial components and nuclear DNA, besides inducing the oxidative modification of proteins and the activation of different signaling pathways [56]. We have examined whether the control of oxidative stress could be involved in the protective effects of CM and extracellular vesicles observed in OA osteoblasts. The results of our analysis indicate that CM, MV, and EX from ASC significantly downregulate the mitochondrial membrane changes and oxidative stress induced by IL-1*β*, thus providing a plausible mechanism to inhibit cellular senescence. Mitochondrial ROS are linked to senescence through nuclear DNA damage [57]. The phosphorylation of H2AX following DNA double-strand breaks increases with age and may be a biomarker for human morbidity in age-related diseases [28]. We found that CM and extracellular vesicles from ASC are able to reduce DNA damage as shown by a lower accumulation of *γ*H2AX foci which may be a consequence of oxidative stress control.

As osteoblasts play an important role in the regulation of cartilage metabolism and bone remodeling, the correction of the abnormal cell metabolism may offer novel therapeutic approaches for joint degradation. Further research into the mechanisms by which senescence of different articular cells contributes to OA is needed to uncover novel targets useful to prevent or treat this condition.

In conclusion, we have shown that CM and extracellular vesicles from ASC downregulate inflammation and oxidative stress which may mediate antisenescence effects in OA osteoblasts. Our data also indicate that MV and EX from ASC are responsible for the paracrine effects of these cells and suggest the interest of these extracellular vesicles to develop new treatments for joint conditions.
